# 3D Bulk Metamaterials with Engineered Optical Dispersion at Terahertz Frequencies Utilizing Amorphous Multilayered Split‐Ring Resonators

**DOI:** 10.1002/advs.202405378

**Published:** 2024-07-08

**Authors:** Ying Huang, Takanori Kida, Shun Wakiuchi, Taiyu Okatani, Naoki Inomata, Yoshiaki Kanamori

**Affiliations:** ^1^ Department of Robotics Tohoku University Sendai Miyagi 980‐8579 Japan

**Keywords:** 3D metamaterial, bulk metamaterial, engineering dispersion, metal‐insulator‐metal structure, split‐ring resonator, terahertz waves

## Abstract

A 3D bulk metamaterial (MM) containing amorphous multilayered split‐ring resonators is proposed, fabricated, and evaluated. Experimentally, the effective refractive index is engineered via the 3D bulk MM, with a contrast of 0.118 across the frequency span from 0.315 to 0.366 THz and the index changing at a slope of 2.314 per THz within this frequency range. Additionally, the 3D bulk MM exhibits optical isotropy with respect to polarization. Moreover, the peak transmission and optical dispersion are tailored by adjusting the density of the split‐ring resonators. Compared to reported conventional approaches for constructing bulk MMs, this approach offers advantages in terms of the potential for large‐scale manufacturing, the ability to adopt any shape, optical isotropy, and rapid optical dispersion. These features hold promise for dispersive optical devices operating at THz frequencies, such as high‐dispersive prisms for high‐resolution spectroscopy.

## Graphical abstract

Inspired by natural isotropic materials, a bulk metamaterial composed of amorphous multilayered split‐ring resonators is proposed to manipulate the refractive index at terahertz frequencies. Experimental results demonstrate a refractive index variation of 2.314 per terahertz around 0.34 THz incidence frequency. This work exhibits optical isotropy, allows flexible shape design, and supports large‐scale manufacturing, providing benefits for dispersive terahertz devices.

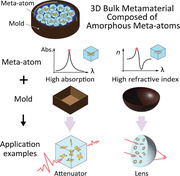

## 1 Introduction

Terahertz (THz) waves have attracted significant attention in recent decades owing to their potential applications in a wide range of fields, such as nondestructive inspection,^[^ ^1^ ^]^ sensing,^[^ ^2^ ^]^ and imaging,^[^ ^3^ ^]^ and these applications can benefit various sectors, including the medical, environmental, and biotechnological sectors.^[^ ^4^, ^5^ ^]^ With the ever‐increasing interest in sixth‐generation (6G) wireless communications, the frequency range of 0.2–0.5 THz, which was proposed as a candidate waveband at the World Radiocommunication Conference 2019,^[^ ^6^ ^]^ has been rapidly developed and become a popular waveband.^[^ ^7^, ^8^, ^9^, ^10^ ^]^ To implement THz technologies in actual communication systems, devices that can optically manipulate THz waves, such as power attenuators and frequency‐selective filters, are indispensable. Most natural materials weakly interact with THz waves and are unsuitable for use in THz devices. Alternatively, metamaterials (MMs), which are artificial materials capable of exhibiting extraordinary optical properties, are potential candidates. MMs are composed of periodic subwavelength meta‐atoms, and the desired properties can be achieved by designing the shape and dimensions of the functional meta‐atom. Since MMs were first proposed by Veselago in 1968,^[^ ^11^ ^]^ they have been extensively developed to control light–matter interactions.^[^ ^12^, ^13^, ^14^, ^15^, ^16^, ^17^, ^18^, ^19^, ^20^, ^21^, ^22^ ^]^ Furthermore, MMs can be combined with other technologies, such as microelectromechanical systems (MEMS),^[^ ^23^, ^24^, ^25^, ^26^ ^]^ phase‐change materials,^[^ ^27^ ^]^ and 2D materials,^[^ ^28^ ^]^ to broaden their applications in optics.

Although MMs can effectively manipulate THz waves, a critical issue must be addressed before they can be applied to practical optical devices in real‐world scenarios: To achieve adequate performance, a sufficiently long interaction path with the incident waves, rather than an interaction confined to a single layer of in‐plane MMs, must be ensured. Consequently, 3D bulk MMs, in which meta‐atoms are stacked in the thickness direction, have been developed.^[^ ^29^ ^]^ One approach to creating bulk MMs involves stacking multiple layers of meta‐atom arrays using lithography.^[^ ^30^, ^31^ ^]^ Liu et al.^[^ ^32^ ^]^ successfully stacked up to five layers of meta‐atoms using stacked electron‐beam lithography, while Valentine et al.^[^ ^33^ ^]^ achieved 21 alternating silver and magnesium fluoride films through focused ion‐beam milling lithography. However, the thickness of reported bulk MMs remains significantly smaller than the operating wavelength. Gansel et al.^[^ ^34^ ^]^ reported a uniaxial helix MM fabricated through direct laser writing (DLW), in which the footprint and height of the 3D MM sample were limited by the DLW lithography process itself. Nonetheless, large‐scale bulk samples have not yet been achieved. Additionally, the functionality of MMs is limited to specific polarizations, with their anisotropic responses complicating full electromagnetic characterization and hindering their use in devices. Membrane projection lithography offers a promising approach for creating 3D bulk MMs with micrometer‐scale characteristic dimensions and arbitrary cavity shapes, which is a crucial step toward achieving isotropic MMs. Burckel et al.^[^ ^35^ ^]^ demonstrated magnetic coupling to vertically oriented split‐ring resonators (SRRs), but thus far, researchers have only shown MM structures with single functional layers. Additionally, this approach presents technological challenges. Therefore, significant hurdles remain in developing techniques for creating large‐scale 3D isotropic MMs.

**Figure 1:**
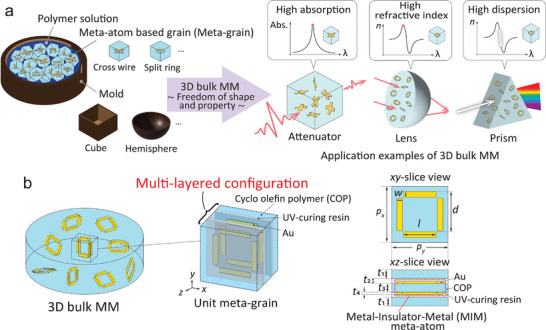
a) Conceptual schematic of the proposed 3D bulk metamaterial (MM) with amorphous metal structures. Left: Principle of the construction of the 3D bulk MM. The meta‐atom based grains (meta‐grain) mixed within a cylinder mold containing polymer solution. Meta‐atoms can have different shapes such as cross wire, split ring, etc., while molds can also vary in shape such as cube, hemisphere, etc. This allows for the creation of custom 3D bulk MMs by altering meta‐atoms and molds. Right: Application examples of different 3D bulk MMs. First: a power attenuator using the meta‐atom with a high absorption. Second: a short‐focus lens at a frequency where the refractive index is high when using the meta‐atom with a high dispersion. Third: a prism at frequencies where the refractive index fluctuates dramatically. Abs.: Absorption. *n*: Real part of the refractive index. *λ*: Wavelength. b) Geometry design of the 3D bulk MM and meta‐grains. Left: Cylinder‐shaped 3D bulk MM. Middle: Unit meta‐grain of the bulk MM. Right: Dimension parameters of the meta‐atom. *w*: Width of the cut wire. *d*: Distance between two parallel wires. *l*: Length of the wire. *t*_1_ to *t*_4_: Thickness of the front and rear COP layers, UV‐curing resin, inside COP layer, gold SRR. *p_x_* and *p_y_*: Side length of the meta‐grain in *x*‐ and *y*‐direction. The value of these parameters are listed in Table 1.

Inspired by the amorphous characteristics of isotropic materials found in nature, we propose a 3D bulk MM configured with amorphous meta‐atoms. Natural isotropic materials typically possess a homogeneous internal structure that is consistent in all directions, resulting in properties that are also uniform in all directions. By employing a similar homogeneous amorphous structure and substituting natural atoms with meta‐atoms, we anticipate that the 3D bulk MM can also demonstrate isotropic properties. **Figure** **1**a illustrates the concept of our proposal. To achieve a homogeneous amorphous structure, we use a polymer with high transmission in the THz band and ease of processing as a carrier to shape the 3D bulk MM. First, metal structures are encapsulated in the polymer to form small grains, with each grain containing a functional meta‐atom, referred to as a meta‐grain in this work. The metal structure can be patterned into desired shape by changing the design of the mask pattern, such as cross wire, split ring, etc. These meta‐atom‐based meta‐grains are then amorphously mixed within a mold containing a polymer solution and solidified to form the 3D bulk MM. The shape of the mold determines the shape of the 3D bulk metamaterial. Various molds like cubes, hemispheres, etc., allow us to create the 3D bulk MM in any desired shape. The final shape of the 3D bulk MM is constrained only by the size and shape of the mold, thus allowing large‐scale manufacturing. Since the meta‐atoms are randomly dispersed within the 3D bulk MM, we expect them to interact with incident waves with different polarizations, leading to optical isotropy. Similar to natural materials, whose properties are determined by the properties of their constituent atoms, the properties of the nature‐inspired 3D bulk MM are influenced by the properties of the unit meta‐atom, allowing for a wide range of potential applications. For instance, by using meta‐atoms with high absorption, the 3D bulk MM can attenuate transmission power, acting as a power attenuator. Alternatively, by using meta‐atoms with high optical dispersion, at a frequency where the refractive index is high, the 3D bulk MM can serve as a short‐focus lens; while in frequency ranges where the refractive index varies significantly, the 3D bulk MM can separate white light into its constituent spectral colors, functioning as a prism. This means that by utilizing the same meta‐atoms, the 3D bulk MM exhibits diverse application potentials across different frequencies, showcasing its multifunctional capabilities. Unlike conventional approaches for creating 3D bulk MMs, our method enables flexible shape‐free fabrication of large‐scale 3D bulk MMs by employing various molds; moreover, this method is polarization‐independent since the functional meta‐atoms are randomly dispersed. A preliminary demonstration of this proposed technology was reported in our prior work,^[^ ^36^ ^]^ in which a 1.6‐mm‐thick 3D bulk MM was constructed, and optical isotropy was confirmed. However, the refractive index of the bulk MM fluctuated with a low slope around the resonant frequency, limiting its application in devices requiring rapid dispersion, such as high‐resolution spectroscopy. Thus, a research gap remains in the development of 3D bulk MMs capable of dramatically engineering the optical dispersion.

Metal–insulator–metal‐structured meta‐atoms (MIM meta‐atoms) have gained popularity due to their strong coupling with incident electromagnetic waves, as electromagnetic fields can be confined not only in‐plane but also out‐of‐plane, i.e., between top and bottom metal structures.^[^ ^37^, ^38^, ^39^, ^40^, ^41^, ^42^, ^43^, ^44^ ^]^ Therefore, the utilization of MIM meta‐atoms may offer a solution for significant dispersion engineering. In this study, for the first time, we introduced the concept of a multilayered meta‐grain based on an MIM meta‐atom, aiming to achieve a 3D bulk MM with rapid optical dispersion. Experimentally, we demonstrated a refractive index contrast of 0.118 around the resonant frequency of 0.34 THz, with the refractive index changing at a slope of 2.314/THz. This slope is 1.25 times larger than that reported in ref. [36] Moreover, the dispersion slope can be controlled by adjusting the meta‐atom density. The MIM meta‐atom comprised cyclo olefin polymer (COP) sandwiched between two gold SRRs. The manufacturing processes for forming the 3D bulk MM consisting of multilayered meta‐grains were established and explained in detail. This work introduces a technique that differs from conventional methods for constructing 3D bulk MMs, offering advantages such as the potential for large‐scale production, design freedom in the shape, optical isotropy, and rapid dispersion. These features hold promise for devices such as high‐resolution spectroscopic prisms.

## 2 Design and Calculations

Figure 1b schematically depicts the proposed 3D bulk MM formed using a cylindrical mold. Within the 3D bulk MM, numerous unit metal structures were dispersed in random orientations. As mentioned earlier, this configuration was achieved by utilizing meta‐grains. Each unit meta‐grain contained an MIM‐structured meta‐atom comprising COP sandwiched between two SRRs. The front and back sides of the meta‐atom were surrounded by two layers of COP, which served as support layers, with UV‐curable resin used as the adhesive. The SRR was composed of four gold rods arranged in pairs orthogonal to each other to form a split ring. Each adjacent pair of rods had identical splits, and the structure was symmetrical in both the *x*‐ and *y*‐directions. The dimensions of the MIM meta‐atom were determined through parametric sweeping calculations to obtain resonances at frequencies ranging from 0.2 THz to 0.35 THz, as listed in **Table** **1**.

**Table 1:** Designed and fabricated dimensions of the meta‐grains.

Parameter	*l*	*d*	*w*	*p_x_*	*p_y_*	*t*_1_	*t*_2_	*t*_3_	*t*_4_
Design [µm]	256.0	320.0	40.0	406.0	406.0	100.0	25.0	50.0	0.1
Fabrication [µm]	256.1	319.6	40.1	572.0	605.0	100.0	25.0	50.0	0.1

The simulation was conducted using CST MICROWAVE STUDIO (CST Studio Suite, Dassault Systèmes S.E.) based on the finite integration technique. The *S*‐parameters were obtained from the simulation software, and then, the transmission was calculated by *T* = |*S*_21_|^2^. In the simulation model, a pulsed plane wave was normally incident. The front surface of the cell was set as the launch and the *S*_11_ monitor, while the rear surface was set as the *S*_21_ monitor. The other four sidewalls were set as unit cell boundaries. The material properties were defined as follows: Au had a permeability of 1 and an electrical conductivity of 4.561 × 10^7^ S m^−1^; the UV‐curable resin had a permeability of 1, a permittivity of 2.4, and a loss tangent of 0.021; and COP had a permeability of 1, a permittivity of 2.34, and a loss tangent of 0.021. The frequency was swept from 0.2 THz to 0.5 THz with a resolution of 6 GHz.

**Figure 2:**
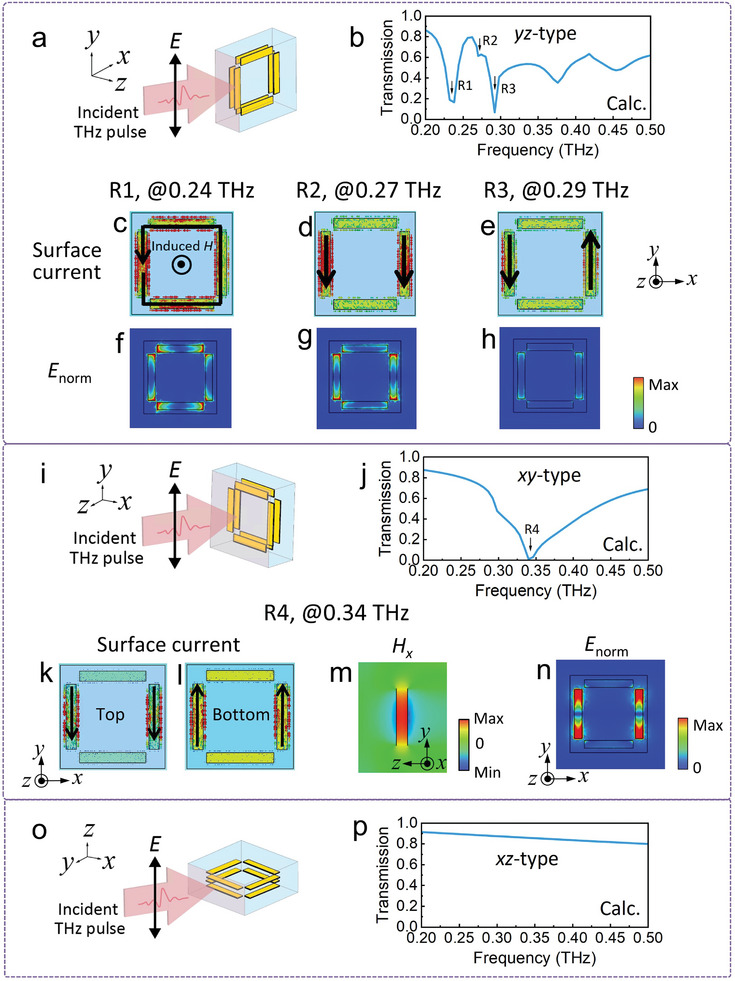
a–h) Schematic and calculations of the meta‐atom array with the *yz*‐type orientation. As the calculated results are identical for the top and bottom SRRs, only the top SRR is shown here. i–n) Schematic and calculations of the meta‐atom array with the *xy*‐type orientation. o,p) Schematic and calculated transmission of the meta‐atom array with the *xz*‐type orientation. The (c–h,k,l,n) slice views were located on the inner surface of the SRR, while the (m) view was located on the cross‐section at the middle of the gold rod aligned with the incident polarization of *E*. The magnitude of the electric field, denoted as *E*_norm_, was defined as $E_{\mathrm{norm}}\;=\;\sqrt{\left(|E_{x}{|}^{2}+\;\left|E_{y}{|}^{2}\;+\;\right|E_{z}{|}^{2}\right)}$.

The 3D bulk MM was filled with meta‐atoms oriented in various directions. To predict the optical response of the 3D bulk MM, the calculation method should account for these various orientations. To approximate the random orientation of the meta‐grains, we assumed the meta‐grain rotates in increments of 90° around three axes (i.e., *x*‐, *y*‐, and *z*‐axis), resulting four basic orientations for each axis: rotation angle was 0°, 90°, 180°, and 270°. Due to the symmetry of the SRR along the *x*‐ and *y*‐axes, freely combining these basic orientations across the three axes yields three orientation types: *yz*‐type, *xy*‐type, and *xz*‐type. See Figure S1 (Supporting Information) and related explanation for more information about the orientation types. These orientations along with the incident waves are schematically illustrated in **Figure** **2**a,i,o. In these orientations, the *yz*‐plane, *xy*‐plane, and *xz*‐plane of the meta‐atom were perpendicular to the incidence wave vectors. We then calculated the optical response of the meta‐atom array with these three orientation types to analyze the resonant modes excited for each of them.

Figure 2b plots the calculated transmission of the meta‐atom array with the *yz*‐type orientation. In the frequency range of interest, from 0.2 THz to 0.35 THz, three distinct resonance dips appeared in the transmission spectrum, labeled R1, R2, and R3. The surface currents on the SRRs at these frequencies were calculated to elucidate the mechanisms of these resonant modes. For resonant mode R1 at 0.24 THz, a circulating current was induced by the external electromagnetic field (Figure 2c), and it generated an induced magnetic dipole moment parallel to the external magnetic field (see Figure S3c, Supporting Information for the calculated *H*‐field of the SRR), indicating that the corresponding transmission dip resulted from a magnetic resonance of the SRRs.^[^ ^45^ ^]^ For resonant mode R2 at 0.27 THz, the pair of gold rods parallel to the incident electric polarization acted as a pair of electric dipoles, producing a dipolar current flowing in the same direction (Figure 2d). This observation indicates that an electric dipole resonance contributed to the corresponding transmission dip. For resonant mode R3 at 0.29 THz, the pair of gold rods parallel to the incident electric polarization behaved as two opposite electric dipoles, generating a current that flowed in the opposite direction (Figure 2e), indicating that the electric dipole resonance was responsible for the corresponding transmission dip. In all these resonant modes, the energy was distributed within the SRR, with no scattering in the surrounding medium, suggesting that the meta‐atom can confine the energy without coupling to neighboring meta‐atoms (Figure 2f–h). These results indicate that the designed meta‐atom is suitable for use in 3D bulk MMs because each meta‐atom independently operates. Figure 2j plots the calculated transmission of the meta‐atom array with the *xy*‐type orientation. A broad valley was observed at the center frequency of 0.34 THz. A surface current loop circulated between the top and bottom gold rods parallel to the incident electric polarization, passing through the middle COP layer (Figure 2k,l), while a strong *H_x_* field was confined between the top and bottom gold rods (Figure 2m). These results provide evidence of excitation of a magnetic resonant mode that leads to a significant dip in transmission spectrum. The energy was also distributed within the SRR, as shown in Figure 2n. Note that compared to the *yz*‐type meta‐atom, the *xy*‐type meta‐atom has much stronger energy confinement. Figure 2p plots the calculated transmission of the meta‐atom array with the *xz*‐type orientation. Since the incident polarization was vertical to the gold rod, no resonance was excited, and the transmission remained high and flat across the entire frequency range.

**Figure 3:**
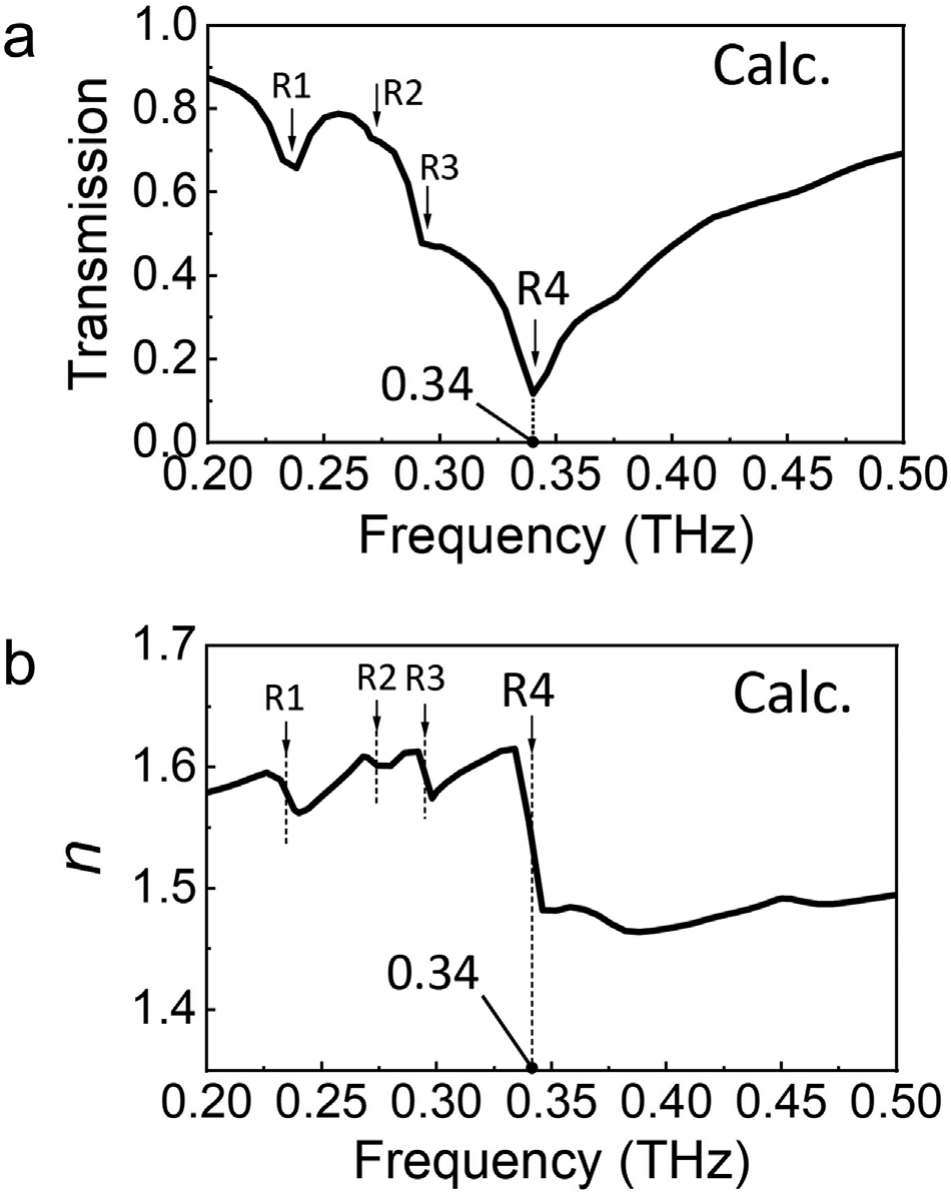
Calculated a) transmission and b) real part of the effective refractive index of the 3D bulk MM.

The optical response of the 3D bulk MM was obtained by considering a combination of meta‐atoms dispersed in the three orientation types. The *S*‐parameters for transmission (*S*_21, orientation type_) and reflection (*S*_11, orientation type_) of three orientation types of meta‐atom array were obtained from simulation in CST software. Then, the *S*‐parameters for transmission (*S*_21, bulk_) and reflection (*S*_11, bulk_) of the 3D bulk MM were obtained for the *S*‐parameters of three types meta‐atom array using the following equations:

(1) $$S_{21,\mathrm{bulk}}=S_{21,\mathrm{yz}-\mathrm{type}}\beta_{\mathrm{yz}-\mathrm{type}}+S_{21,\mathrm{xy}-\mathrm{type}}\beta_{\mathrm{xy}-\mathrm{type}}+S_{21,\mathrm{xz}-\mathrm{type}}\beta_{\mathrm{xz}-\mathrm{type}}$$

(2) $$S_{11,\mathrm{bulk}}=S_{11,\mathrm{yz}-\mathrm{type}}\beta_{\mathrm{yz}-\mathrm{type}}+S_{11,\mathrm{xy}-\mathrm{type}}\beta_{\mathrm{xy}-\mathrm{type}}+S_{11,\mathrm{xz}-\mathrm{type}}\beta_{\mathrm{xz}-\mathrm{type}}$$

where parameter *β* represents the proportion of each type of meta‐atom dispersed in the 3D bulk MM. *β* was a volume‐related parameter and affected the magnitude of the resonance of the 3D bulk MM. See Figure S4 (Supporting Information) and related explanation for more information about the parameter *β*. As the meta‐grain had a cuboid form, the side lengths along the *x*‐axis and *y*‐axis were equal and greater than the side length along the *z*‐axis, making the *xy*‐type the dominant orientation in the final 3D bulk MM. Here, we determined the proportions based on our prior experimental data as follows: *β_yz_*_‐type_ = 18%, *β_xy_*_‐type_ = 62%, and *β_xz_*_‐type_ = 20%. Then, the transmission of the 3D bulk MM could be calculated as *T* = |*S*_21, bulk_|^2^. The real part of the effective refractive index (*n*) was retrieved from the *S*‐parameters (*S*_21, bulk_ and *S*_11, bulk_) by using the method reported in ref. [46]. **Figure** **3** plots the calculated transmission and *n* of the 3D bulk MM. The thicknesses in the simulation model geometries were configured as single layers of meta‐grains. Consequently, the transmission spectrum depicted in Figure 3 shows the response of the 3D bulk MM consisting of a single layer of amorphous meta‐atoms. Four resonant modes (R1 to R4) excited by *yz*‐ and *xy*‐type meta‐atoms can be observed in the transmission spectrum, along with corresponding fluctuations in *n* at the resonant frequencies. The resonant dip excited by mode R4 was more pronounced compared to dips at other frequencies. This is because mode R4 was primarily excited by *xy*‐type meta‐atoms, which was the dominant orientation in the 3D bulk MM. The transmission at the center frequency of mode R4 (0.34 THz) was 0.118, and *n* fluctuated from a maximum of 1.615 at 0.334 THz to a minimum of 1.482 at 0.346 THz. The contrast in *n* (∆*n* = *n*_max_−*n*_min_) was 0.133, and the frequency span (∆*f* = *f*_High_−*f*_Low_) of the index fluctuation was 0.012 THz. The optical dispersion slope, defined as ∆*n*/∆*f*, was 11.08/THz.

## 3 Fabrication

**Figure 4:**
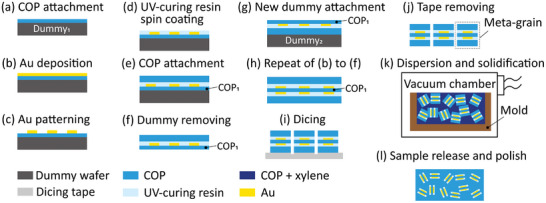
Fabrication processes of the 3D bulk MM.

The fabrication process for the proposed 3D bulk MM is schematically detailed in **Figure** **4**. Initially, a 50‐µm‐thick COP film (Zeonor Film ZF16, Nippon Zeon Co., Ltd.) was adhered to a 4‐inch dummy silicon wafer using polyimide tape (Figure 4a). Subsequently, a 100‐nm‐thick gold film was deposited onto the COP film by sputtering (Figure 4b). The gold film was then patterned into a split‐ring array using photolithography (photoresist: OFPR‐800 34cp, Tokyo Ohka Kogyo Co., Ltd.) and wet etching (etchant: AURUM‐302, Kanto Kagaku Co., Ltd.) processes (Figure 4c). Next, UV‐curable resin (ThreeBond 3017D, ThreeBond Co., Ltd.) was spin‐coated onto the gold pattern to a thickness of 25 µm (Figure 4d), followed by covering of the sample with another 100‐µm‐thick COP film (Figure 4e). The dummy wafer was then removed (Figure 4f), and another dummy wafer was attached to the 100‐µm‐thick COP film (Figure 4g). Subsequently, the steps outlined in Figure 4b–f were repeated to create an MM sheet containing an MIM meta‐atom array with 50‐µm‐thick COP sandwiched between two gold SRRs (Figure 4h). Next, the MIM MM sheet was affixed to dicing tape and diced using a diamond blade with a thickness of 100 µm (R07‐SD500‐BB200‐75 52×0.1A2×40, DISCO Co., Ltd.) (Figure 4i). At this point, meta‐grains were prepared on the dicing tape. Subsequently, the dicing tape was removed, and the meta‐grains were exfoliated from the tape in an acetone solution after weakening the adhesion of the tape. The exfoliated meta‐grains were then rinsed with deionized water and dried in an oven at 75 °C for 30 min (Figure 4j). Next, 0.37 g of a solution containing 35 wt.% COP grains (ZEONEX 480R, Nippon Zeon Co., Ltd.) dissolved in xylene was poured into a cylindrical mold with an inner diameter of 16 mm and a height of 4 mm, followed by manual mixing of 0.31 g of the meta‐grains into the solution. The mold was then placed in a vacuum chamber at room temperature for 12 h to defoam and solidify the solution, thus reconfiguring the dispersed meta‐grains into a 3D bulk MM (Figure 4k). Finally, the 3D bulk MM was removed from the mold, and the surfaces were polished to reduce optical scattering (Figure 4l).

3D bulk MMs with various meta‐grain densities were fabricated by changing the mass proportion of meta‐grains dispersed in the solution. To lower the mass proportion (*γ*), the meta‐grains were mixed with COP grains before being added to the solution. Specifically, for pure meta‐grains without COP grains, *γ* was defined as *γ* = 100%; for meta‐grains mixed with COP grains in a mass ratio of 1:1, *γ* was defined as *γ* = 50%; and for meta‐grains mixed with COP grains in a mass ratio of 1:3, *γ* was defined as *γ* = 25%. Then, 0.31 g of the different grain mixtures was mixed into 0.37 g of solution, following the process shown in Figure 4k. After solidification, the density (*D*) of the meta‐grains in the 3D bulk MM was defined as *D* = *γ*, i.e., *D* = 100% for meta‐grains with a *γ* of 100%, *D* = 50% for meta‐grains with a *γ* of 50%, and *D* = 25% for meta‐grains with a *γ* of 25%.

**Figure 5:**
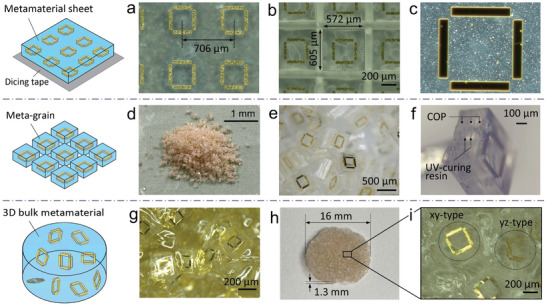
a–c) Fabricated MM sheet, d–f) meta‐grains, and g–i) 3D bulk MM. Insets display schematic diagrams of optical images for each row to aid understanding.

**Figure** **5** shows optical images and schematic illustrations of the fabricated MM sheet (Figure 5a–c), meta‐grains (Figure 5d–f), and 3D bulk MM with a density *D* of 100% (Figure 5g–i). Figure 5a,b show magnified top views of the MM sheet on dicing tape before and after dicing, respectively. The distance between adjacent SRR centers in the initial MM sheet was 706 µm (Figure 5a). The distance was designed to exceed the side length of 406 µm of the meta‐grains to accommodate the effects of the dicing width and dicing error. Post‐dicing, the measured side lengths of a one‐unit cell were *p_x_* = 572 µm and *p_y_* = 605 µm (Figure 5b). By optimizing the dicing conditions, the experimental values of *p_x_* and *p_y_* can be brought closer to the designed values. In our simulation, we set experimental values for *p_x_* and *p_y_*. See Figure S5 (Supporting Information) and related explanation for more information of the simulation model. Figure 5c shows a zoomed‐in view of a one‐unit cell, revealing perfect overlap between the top and bottom SRRs, indicating precise alignment of the two SRR layers. Figure 5d shows meta‐grains peeled off from the dicing tape. Each meta‐grain contained one SRR‐based MIM meta‐atom (Figure 5e). The meta‐grain featured a multilayered configuration consisting of three COP layers separated by two UV‐curable resin layers, with the middle COP layer sandwiched between two gold SRR layers (Figure 5f). The dimensions of the meta‐grains are listed in Table 1. Figure 5g shows an enlarged view of the meta‐grains mechanically mixed into the solution, in which the meta‐grains were randomly dispersed. Following solidification and polishing, a pie‐shaped 3D bulk MM with a diameter of 16 mm and a thickness of 1.3 mm was successfully fabricated (Figure 5h). A magnified view of the 3D bulk MM is shown in Figure 5i. Different types of meta‐atoms were dispersed in the 3D bulk MM as anticipated. As an example, *xy*‐ and *yz*‐type SRRs can be observed in the area pictured in Figure 5i.

## 4 Results and Discussion

### 4.1 Results

The transmission characteristics of the fabricated 3D bulk MM were measured with a THz time‐domain spectrometer (THz‐TDS) (TeraProspector, Nippo Precision Co., Ltd.). *y*‐polarized THz pulse radiation passed through a 10 mm diameter aperture and was normally incident on the sample under test. The frequency range of the incident radiation spanned from 0.2 to 0.5 THz with an interval of 6 GHz. The measured THz transient electric field was subjected to fast Fourier transformation to yield the frequency‐domain spectrum, and the output complex spectrum was separated into the intensity (*A*(*f*)) and the phase (*φ*(*f*)), where *f* is the frequency. In this study, an air background was measured as a reference, and the transmission of the sample was normalized to the air reference according to $T\left(f\right)\;=\;\frac{A{\left(f\right)}_{\mathrm{Sample}}}{A{\left(f\right)}_{\mathrm{air}}}$. The real part of the refractive index (*n*(*f*)) was extracted from the measured phase delay of the sample using the formula $n\left(f\right)\;=\;\frac{\varphi {\left(f\right)}_{\mathrm{Sample}}-\varphi {\left(f\right)}_{\mathrm{air}}}{k_{0}d}$, where *k*_0_ denotes the wavenumber in vacuum and *d* denotes the thickness of the sample.

**Figure 6:**
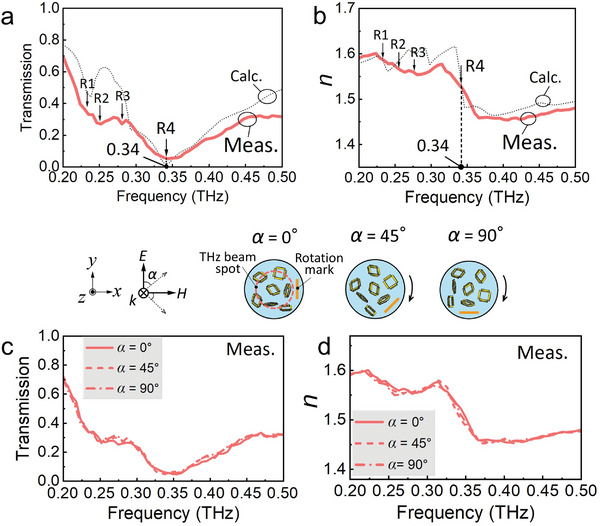
Measured optical response of the fabricated 3D bulk MM: a) transmission (red solid curve) and b) refractive index (red solid curve). The calculations are plotted as black dashed curves for comparison. c) Transmission and d) refractive index at various incident angles. The middle inset schematically illuminates the measurement at various polarization angle to aid understanding. A tape was fixed on the 3D bulk MM as a location mark, and the rotation angle of the 3D MM was confirmed using this mark.

**Figure** **6**a presents the measured transmission of the fabricated 3D bulk MM with a *D* of 100%, along with the calculated data for comparison. Since the calculation model thickness was set as a single layer of meta‐grains of 668 µm (see Figure S5, Supporting Information for more information of this parameter), the calculated transmission was adjusted by raising it to the 1.95th power to match the transmission of the fabricated 3D bulk MM, which had a thickness of 1.3 mm, for comparison. As predicted by the calculations, four dips were observed in the measured spectrum at 0.24, 0.25, 0.28, and 0.34 THz. This confirms that the resonances caused by *yz*‐ and *xy*‐type meta‐atoms were observed in the experiments. The center frequency of the primary resonant mode R4 well matched the calculation result. The measured transmission (*T*_c_) at 0.34 THz was 0.062. Figure 6b shows the measured *n* of the fabricated 3D bulk MM along with the calculated data. At the resonant frequencies of modes R1 to R4, fluctuations were observed in the measured *n* curve, indicating that these dips in the transmission spectrum resulted from changes in *n*. Around the resonant frequency of mode R4, *n* decreased from 1.577 at 0.315 THz to 1.459 at 0.366 THz. Notably, a contrast ∆*n* of 0.118 was obtained across a frequency span ∆*f* of 0.051 THz, and *n* changed with a slope of 2.314/THz within this frequency span.

The measured transmission dip corresponding to mode R4 was wider than that in the calculation, in other words, the resonance dip had a smaller quality factor (*Q*‐factor) compared to theoretical predictions. Since *n* was retrieved from the *S*‐parameters in the calculation, the narrower dip in transmission led to a larger optical dispersion slope than observed experimentally. This discrepancy might be attributed to optical scattering caused by air bubbles within the fabricated 3D MM, resulting in a reduced *Q*‐factor for the transmission dip. These losses were not quantitatively accounted for in the calculations. Additionally, transmission dips corresponding to R1 and R2 were smaller in measurements than those calculated. This might be due to non‐uniform distribution of meta‐atoms within the actual 3D bulk MM, where atom‐to‐atom spacing was uneven compared to idealized assumptions in calculations. As countermeasures to reduce these discrepancies, optimizing the defoaming process could minimize air bubbles and reduce losses. Utilizing a mixing machine instead of manual tweezers for meta‐grain dispersion might achieve more uniform distribution. Despite these challenges, calculations successfully identified all resonant modes observed in experimental data. Moreover, resonant frequencies of interest and the overall transmission spectrum closely matched experimental measurements, suggesting that calculations can effectively guide experimental investigations. We fabricated another sample under the identical fabrication conditions, and a good reproducibility of samples was confirmed (see Figure S6, Supporting Information and related explanations for more information).

To verify the polarization independence of the optical response, after the initial measurement, the 3D bulk MM was rotated clockwise to angles (*α*) of 45° and 90° with respect to the polarization of the incident light (see the inset schematics in Figure 6) and subsequently measured. Figure 6c,d display the transmission and *n* of the 3D bulk MM measured at various rotation angles. Here, the transmission spectrum and *n* curve for *α* = 0° in Figure 6a,b were replotted. In all the transmission spectra and *n* curves, four resonance dips were consistently observed, indicating that the 3D bulk MM exhibited polarization independence.

**Figure 7:**
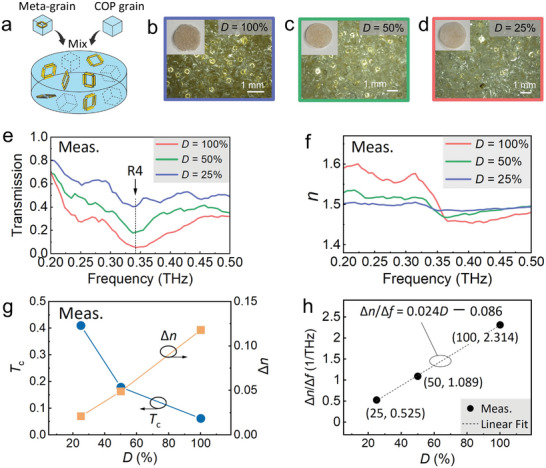
a) Schematic of the fabrication method. b–d) Magnified views of the fabricated 3D bulk MMs with various *D* values of 100%, 50%, and 25%. The insets show overviews of the 3D bulk MMs. Measured optical response of the fabricated 3D bulk MMs with various *D* values: e) transmission, f) refractive index, g) peak transmission and contrast in n as a function of *D*, h) optical dispersion slope as a function of *D*.

3D bulk MMs with *D* values of 50% and 25% were fabricated by mixing meta‐grains and COP grains before adding them to the solution, as described in the Fabrication section (see **Figure** **7**a for a schematic illustration). Magnified views of the fabricated 3D bulk MMs with *D* values of 100%, 50%, and 25%, along with global views as insets, are shown in Figure 7b–d, respectively. As *D* decreased, the 3D bulk MM became transparent due to the reduction in the amount of the gold pattern. The density of the meta‐atoms was confirmed to be reduced, and each 3D bulk MM contained various types of meta‐atoms. The measured transmission spectra of the 3D bulk MMs are plotted in Figure 7e. Fluctuations were observed in all the transmission spectra, indicating that the different resonant modes did not disappear as *D* decreased from 100% to 25%. The dip caused by mode R4 exhibited an increase in *T*_c_ as *D* decreased, with *T*_c_ measured as 0.179 for *D* = 50% and 0.410 for *D* = 25%. Figure 7f plots the measured *n* curves of the 3D bulk MMs. As *D* decreased, ∆*n* decreased to ∆*n* = 0.049 for *D* = 50% and ∆*n* = 0.021 for *D* = 25%. With decreasing meta‐grain density, *T*_c_ increased due to the reduced gold content, while ∆*n* decreased due to the reduced number of meta‐atom resonators (Figure 7g), indicating that this trade‐off should be considered when designing 3D bulk MMs depending on their applications. The dispersion slope linearly decreased with decreasing *D*, with ∆*n*/∆*f* = 1.089 for *D* = 50% and ∆*n*/∆*f* = 0.525 for *D* = 25%, and this tendency could be predicted by the linear fit ∆*n*/∆*f* = 0.024D − 0.086 (Figure 7h). These results indicate that the optical performance of the 3D bulk MM can be controlled by adjusting the density of the meta‐grains. **Table** **2** summarizes the optical properties of the 3D bulk MMs with various *D* values.

**Table 2:** Optical properties of 3D bulk MMs with various *D* values (@ resonant mode R4).

*D*	*T*_c_	∆*n*	∆*n/*∆*f* [1/THz]	Index fluctuation range *n_max_ ≈ n_min_*	Frequency span of the index change *f_Low_ ≈ f_High_* [THz]
100%	0.062	0.118	2.314	1.577–1.459	0.315–0.366
50%	0.179	0.049	1.089	1.518–1.469	0.315–0.360
25%	0.410	0.021	0.525	1.506–1.485	0.315–0.355

### 4.2 Discussion

The 3D bulk MM proposed in this study, as an artificial material, can be used to engineer the dispersion with a rapid slope, with *n* changing at a rate of 2.314/THz. Compared to the rate of 1.849/THz reported in ref. [36], this work represents a 25 percentage point increase. Such *n* modulation makes the 3D bulk MM advantageously beneficial for applications such as prisms or lenses. The sharp variation of refractive index over a narrow frequency range enables the 3D bulk MM to spectrally separate light into its constituent waves, and this capability to split narrow‐band light results in high‐resolution dispersion of prism. At the specific frequency where *n* is high, the 3D bulk MM can function as a short‐focus lens. This work highlights several advantages over conventional approaches. Unlike components using natural materials, this work enables unrestricted control over *n*. Conventional meta‐surface components are flat optics, the phase profile of the components needs to be folded every 2π. Therefore, their design frequency is typically fixed, and deviations from this frequency may lead to higher‐order diffracted waves. In contrast, our bulk MM do not have phase folding, so they are essentially free of the above issues and may be able to realize more efficient components. A fabrication process for bulk MMs containing MIM‐structured meta‐atoms has been established. By using this scheme and other types of meta‐atoms, the properties of MMs can be tailored to meet specific requirements.

Based on the prototype demonstration of the proposed technology, the issue of low transmission at resonant frequencies needs to be addressed before industry adoption. One potential solution to improve the transmission is to replace the UV‐curable resin with a highly transparent material. Alternatively, meta‐atoms with both high transmission and a high refractive index, as reported in ref. [47], could be utilized in conjunction with the scheme presented in this work. Another approach to address losses is to introduce gain materials into the MM structure, as discussed in refs. [29, 48].

## 5 Conclusion

A 3D bulk MM containing amorphous multilayered metal structures was proposed, fabricated, and evaluated. The experimental results demonstrated that the effective refractive index was engineered via the 3D bulk MM, with a contrast of 0.118 across the frequency span from 0.315 THz to 0.366 THz and the index changing at a slope of 2.314/THz within this frequency range. Additionally, the 3D bulk MM exhibited polarization independence of its optical response. Moreover, the peak transmission and optical dispersion could be tailored by adjusting the meta‐grain density. Compared to conventional methods for constructing 3D bulk MMs, this method offers advantages such as the potential for large‐scale production, optical isotropy, and rapid optical dispersion. These features hold promise for dispersive optical devices operating at THz frequencies.

## Conflict of Interest

The authors declare no conflict of interest.

## Supporting information

Supporting Information

## Data Availability

The data that support the findings of this study are available from the corresponding author upon reasonable request.
