# Supplementary material for: 3D Bulk Metamaterials with Engineered Optical Dispersion at Terahertz Frequencies Utilizing Amorphous Multilayered Split‐Ring Resonators
Source: Adv Sci (Weinh). 2024 Jul 8;11(34):2405378. doi: 10.1002/advs.202405378 (PMC11425637; doi:10.1002/advs.202405378)
Supplement: Supplementary file 1 — Supporting Information [file ADVS-11-2405378-s001.docx]

**Supporting Information**

**3D bulk metamaterials with engineered optical dispersion at terahertz frequencies utilizing amorphous multilayered split-ring resonators**

*Ying Huang*, Takanori Kida, Shun Wakiuchi, Taiyu Okatani, Naoki Inomata, and Yoshiaki Kanamori**

Department of Robotics, Tohoku University, Sendai, Miyagi 980-8579, Japan

*ykanamori@tohoku.ac.jp

*ying.huang.e8@tohoku.ac.jp

To approximate the random orientation of the meta-grains, we assumed the meta-grain rotates in increments of 90° around three axes (i.e., *x*-, *y*-, and *z*-axis), resulting four basic orientations for each axis: rotation angle was 0°, 90°, 180°, and 270° (Fig. S1(a)). Figures S1 (b-d) illustrate these basic orientations for rotations around the *z*-, *x*-, and *y*-axis at four rotation angles, respectively. Due to the symmetry of the SRR along the *x* and *y* axes, rotations around the *z*-axis at different angles yield identical orientations (Fig. S1(b)). Similarly, rotations around the *x*-axis (Fig. S1(c)) and *y*-axis (Fig. S1(d)) show that 0° and 180° rotations correspond to the same orientation, as do 90° and 270° rotations. Combining these basic orientations across the three axes freely yields a total of 4^3 = 64 possible orientations. However, due to the SRR's symmetry, these 64 orientations finally manifest in three types: *xy*-type (Fig. S1(e)), *yz*-type (Fig. S1(f)), and *xz*-type (Fig. S1(g)).

In the actual fabricated bulk MM, orientation possibilities include finer increments of rotation angles, not only 90° we considered in simulation model. Figure S2 shows calculated transmission spectra for a meta-grain array rotated around the *z*-axis at 0°, 15°, and 45° (Fig. S2(a)), and around the *x*-axis at 0°, 15°, 45°, and 75° (Fig. S2(b)). Rotation around the *z*-axis shows no significant variation in transmission spectra due to the SRR's symmetry, indicating the orientation at all angles can be considered as *xy*-type. Rotation around the x-axis reveals distinct resonant dips in transmission spectra for angles *θ_x_* ≤ 45°, which approximate those at *θ_x_* = 0°, classified as yz-type. As the angle increases to *θ_x_* = 75°, the spectra closely resemble those at *θ_x_* = 90°, which is *xz*-type. Therefore, to reduce the computational complexity, rotation angles *θ_x_* ≤ 45° were considered as *yz*-type, while angles *θ_x_* > 45° were considered as *xz*-type.


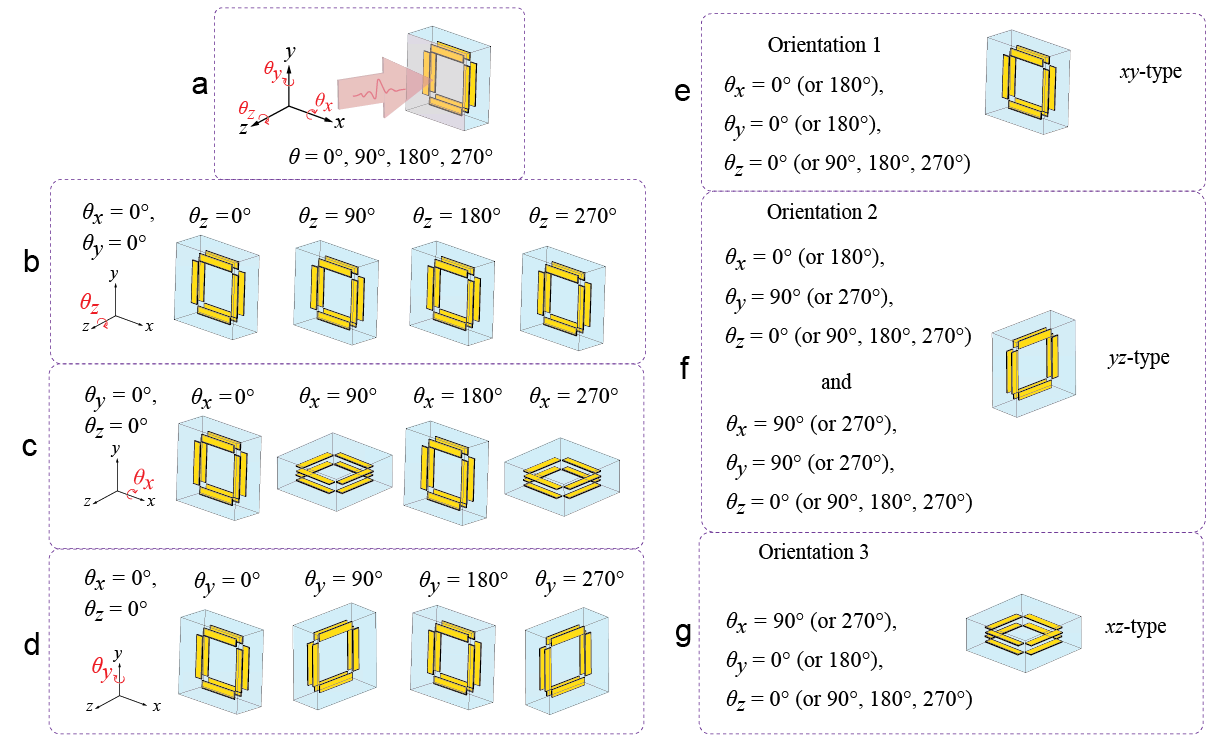


**Figure S1.** Orientations of the meta-grain. (a) The meta-grain was assumed to rotate in increments of 90° around three axes. Basic orientations for rotations around the (b) *z*-, (c) *x*-, and (d) *y*-axis at four rotation angles. Freely combining these basic orientations across the three axes yielding three orientation types: (e) *xy*-type, (f) *yz*-type, and (g) *xz*-type.


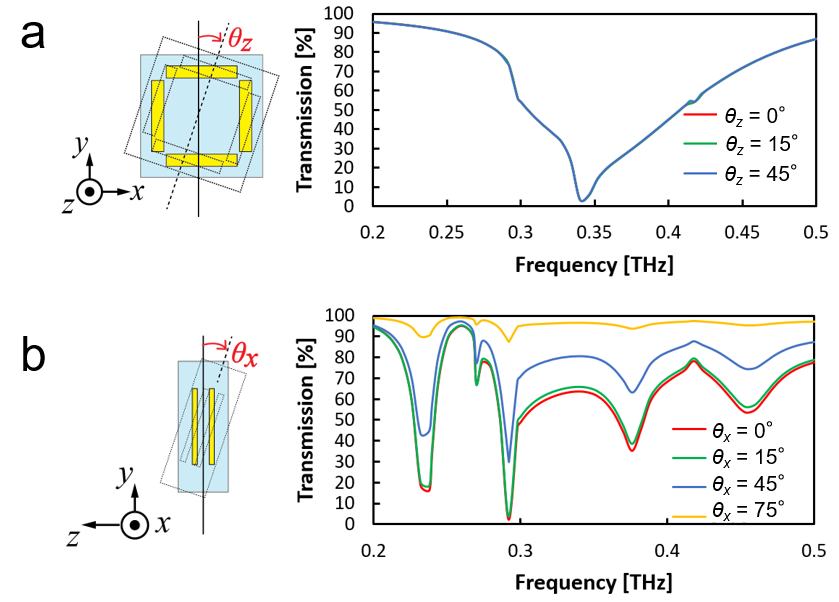


**Figure S2.** Calculated transmission spectra of the meta-atom array that rotated around (a) the *z*-axis at 0°, 15°, and 45°, and (b) the *x*-axis at 0°, 15°, 45°, and 75°. Here, the transmission of *θ_z_* = 0 and *θ_x_* = 0 were higher than that plotted in Figs 2 (j) and 2(b) in the main text, because the loss of the COP was ignored in this calculation.

Figure S3(a) depicts a schematic of the *yz*-type meta-atom along with the incident waves. Figure S3(b) plots calculated transmission spectrum of the meta-atom array. Figure S3(c) shows calculated *H*-fields in *z*-component (*H_z_*) of the SRR at 0.24 THz. An induced magnetic dipole moment parallel to the external magnetic field was observed. Figure S3(d) shows calculated *H_z_* at 0.27 THz. The *H_z_* field concentrated along the pair of gold rods parallel to the incident electric polarization, with respect to an electric dipole induced current in Fig. 2(d) of the text. Figure S3(e) shows calculated *H_z_* at 0.29 THz, where the *H_z_* concentrates along the same pair of gold rods but in opposite directions, which proofs that the rods acted as two opposite electric dipoles.


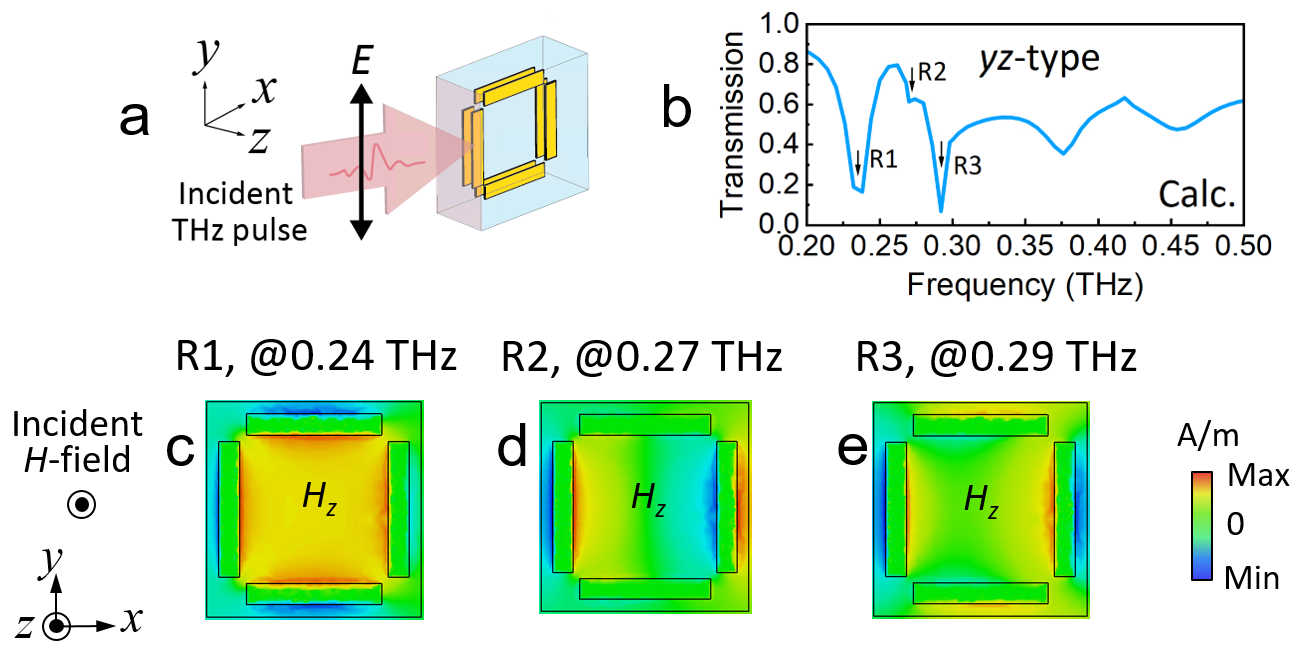


**Figure S3**. (a) Schematic of the *yz*-type meta-atom along with the incident waves. (b) calculated transmission spectrum of the meta-atom array. Calculated *H*-fields in *z*-component (*H_z_*) of the SRR at (c) 0.24 THz, (d) 0.27 THz, and (e) 0.29 THz. As the calculated *H_z_* are identical for the top and bottom SRRs, only the top SRR is shown here.

The parameter *β* was a volume-related parameter. When meta-grains were randomly mixed in a mold, the distribution of each orientation type depended on the side lengths of the meta-grains along the *x*, *y*, and *z* axes. *β* can be controlled during fabrication by designing corresponding side lengths. For example, by setting the side lengths along all three axes of the meta-grain to be equal, i.e., *p_x_* = *p_y_* = the total thickness of the meta-grain, a cubic meta-grain can be achieved, resulting in a *β* ratio of 1:1:1, as demonstrated in our previous work [36]. To map the optical response of the 3D MM across different *β*, we calculated the transmission and n of the 3D bulk MM with *β* ratios of *β*_yz-type_: *β*_xy-type_: *β*_xz-type_ = 1:1:1. These results are plotted in Fig. S4 and compared with those using a *β* ratio of 18:62:20. Resonances excited by different types of meta-atoms were observed, where the magnitude of these resonant dips depended on the *β* value of each type.


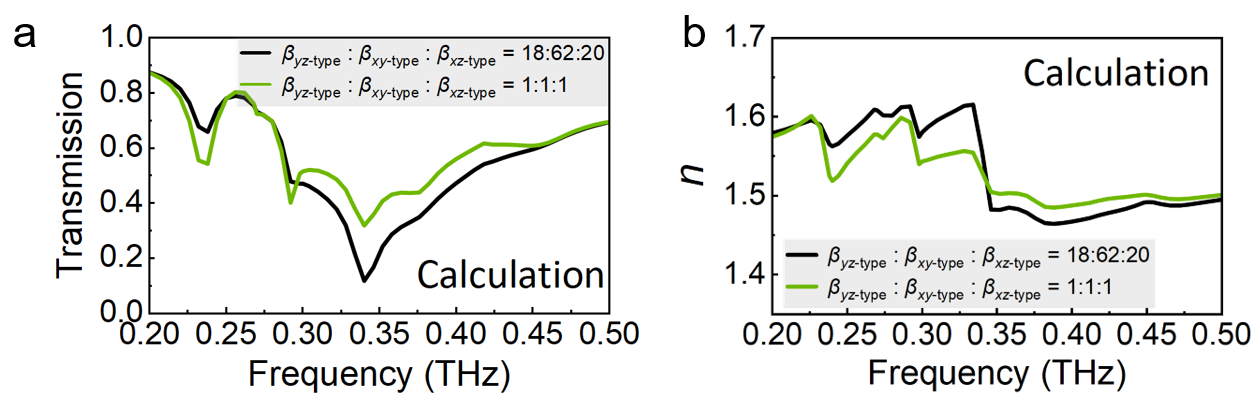


**Figure S4**. Calculated (a) transmission and (b) *n* of the 3D bulk MM with various of *β* ratio.

During fabrication, the meta-grain was mixed in COP solution followed by solidification to form the final bulk MM. Thus, the atom-to-atom spacing in the final bulk MM was larger than the side length *p_x_* and *p_y_* of the meta-grain. In our simulation model, the geometry of the unit cell was built as a cube COP embedding a meta-grain to approximate the real unit cell of the fabricated bulk MM, as shown in Fig. S5. The cube COP had an overall side length *S*, which represents the atom-to-atom spacing of the real bulk MM. The embedded meta-grain had side lengths *p_x_* and *p_y_*. The value of *S* was determined by the volume ratio of the meta-grain within the bulk MM, thus it was related to the side lengths *p_x_* and *p_y_* of the meta-grain. Therefore, parameters such as *p_x_* and *p_y_* influenced the optical response of the bulk MM by affecting the atom-to-atom spacing *S*. In our simulations, we set *S* to the experimental value of 668 μm to accurately model the optical behavior of the 3D MM.


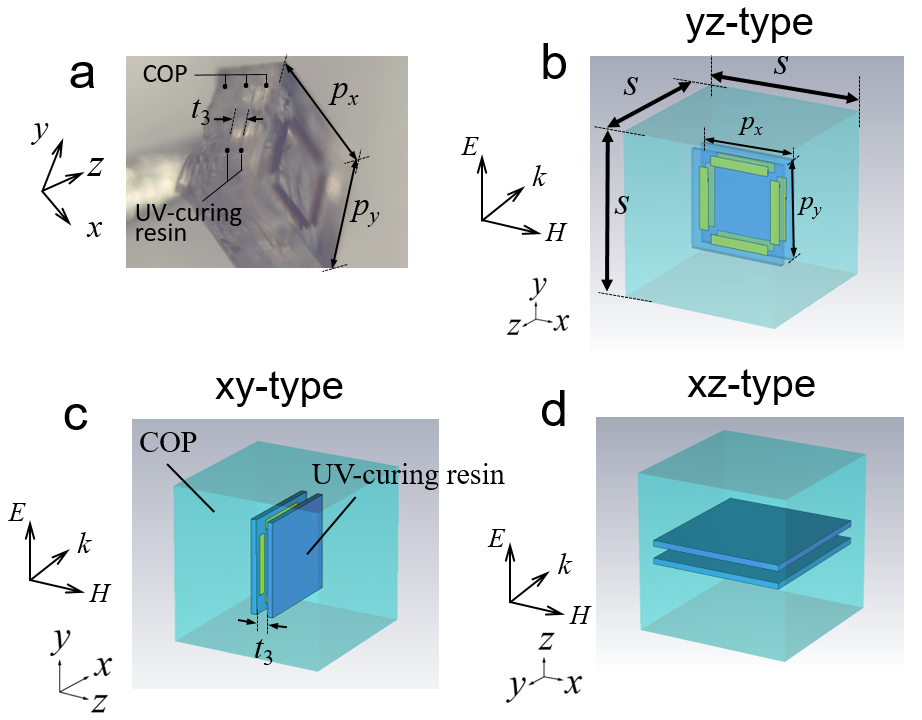


**Figure S5**. (a) Optical image of a fabricated meta-grain with side lengths *p_x_* along the *x*-axis and *p_y_* along the *y*-axis, and thickness *t*_3_ of the inside COP layer. The geometry model of a single unit cell in simulation for meta-atoms oriented in (b) *yz*-type, (c) *xy*-type, and (d) *xz*-type. A cube of COP embedding a meta-grain was used to approximate the real unit cell of the fabricated bulk MM. The cube of COP had an overall side length *S*, while the embedded meta-grain has side lengths *p_x_* and *p_y_*, and thickness *t*_3_ of the inside COP layer.

We fabricated another sample (sample #1) under the identical fabrication conditions. The measured transmission spectrum and n curve are plotted in Figure S6, alongside those of Sample #2 shown in the text. At mode R4 of interest, the resonant frequency remained consistent, and the transmission dip was clearly observed. The fluctuation slope of *n* was not reduced. These results confirm the good stability of the produced samples.


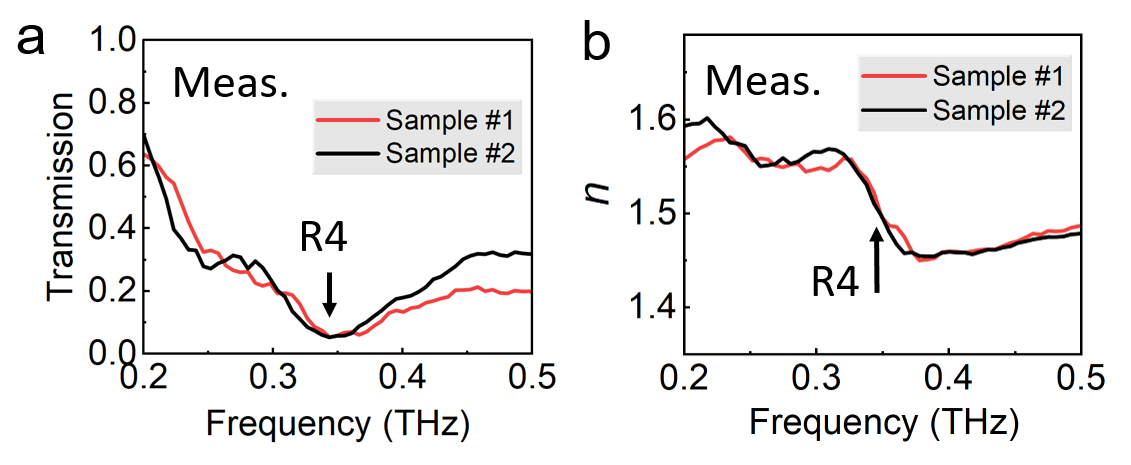


**Figure S6.** Measured (a) transmission and (b) *n* of two samples fabricated with the same condition.
